# Health inequalities in a middle-income country: a systematic review of the Costa Rican case

**DOI:** 10.3389/fpubh.2024.1397576

**Published:** 2024-08-21

**Authors:** Cristina Barboza-Solis, Rolando Herrero, Romain Fantin

**Affiliations:** ^1^Facultad de Odontología, Universidad de Costa Rica, San José, Costa Rica; ^2^Agencia Costarricense de Investigaciones Biomédicas, Fundación INCIENSA, San José, Costa Rica

**Keywords:** health inequalities, Costa Rica, low and middle-income country, systematic review, social determinants of health, health inequities

## Abstract

**Objective:**

This study systematically reviews evidence of socioeconomic health disparities in Costa Rica, a middle-income country, to elucidate the relationship between socioeconomic status and health outcomes.

**Methods:**

Published studies were identified through a systematic review of PubMed (English) and Scielo (Spanish) databases from December 2023 to January 2024, following PRISMA guidelines. Search terms included socioeconomic status, social determinants, social gradient in health, and health inequalities.

**Results:**

Of 236 identified references, 55 met the inclusion criteria. Findings were categorized into health inequalities in mortality (among the general population, infants, and older adults), life expectancy, cause-specific mortality, and health determinants or risk factors mediating the association between the social environment and health. The studies indicate higher mortality among the most disadvantaged groups, including deaths from respiratory diseases, violence, and infections. Higher socioeconomic status was associated with lower mortality rates in the 1990s, indicating a positive social gradient in health (RII = 1.3, CI [1.1–1.5]). Disparities were less pronounced among older adults. Urban areas exhibited concentrated wealth and increased risky behaviors, while rural areas, despite greater socioeconomic deprivation, showed a lower prevalence of risky behaviors. Regarding smoking, people living in rural areas smoked significantly less than those in urban areas (7% vs. 10%). Despite the relatively equitable distribution of public primary healthcare, disparities persisted in the timely diagnosis and treatment of chronic diseases. Cancer survival rates post-diagnosis were positively correlated with the wealth of districts (1.23 [1.12–1.35] for all cancers combined).

**Conclusion:**

The study highlights the existence of social health inequalities in Costa Rica. However, despite being one of the most unequal OECD countries, Costa Rica shows relatively modest social gradients in health compared to other middle and high-income nations. This phenomenon can be attributed to distinctive social patterns in health behaviors and the equalizing influence of the universal healthcare system.

## 1 Introduction

Health is shaped over the life course and is determined by the circumstances in which people are born, grow, live, work, and age (1). According to the World Health Organization, these conditions are molded by political, social, and economic forces (1). The distribution of health determinants between socioeconomic groups creates a socioeconomic gradient in health in the majority of countries (2). Individuals at the bottom of the social hierarchy are more likely to experience unhealthy lives compared to those in the middle, who, in turn, have worse health than those at the top (3). Recognized as systematic, socially produced, modifiable, and unfair (4), the social gradient in health affects a broad range of health indicators, from risk factors to health outcomes (5). The association between measures of socioeconomic status (SES) and diverse health outcomes is a consistent finding in epidemiologic research (6), and continues to be a challenge for both high-income (7) and low- and middle-income countries (LMICs) (8).

Despite recent advances in highlighting social determinants of health (9), research in LMICs remains underrepresented (10). In the case of Latin America and the Caribbean (LAC), shows that lower socioeconomic status (SES) is associated with higher mortality, although research on this topic remains relatively sparse (11, 12). LAC remains one of the most inequitable regions globally, with significant poverty (13), a dual burden of infectious and non-communicable diseases, rapid aging, migration influxes, and weak public health structures for epidemiologic surveillance (8, 14).

For a middle-income country, it is imperative to tackle health inequalities for cost-effective healthcare. These inequalities often result in increased healthcare budgets, a higher proportion of out-of-pocket health expenditures, and increased disability and disease burden, ultimately leading to preventable and premature mortality (15). By reducing socioeconomic disparities in health, our societies move closer to achieving social equity and justice, ensuring a better quality of life and striving for the principle of “leaving no one behind” (16).

Costa Rica, an upper-middle-income country in Central America, has been an OECD member since 2022. With a population of 5 million, mostly urban and concentrated around the capital, 72% live in urban areas and 62% in the metropolitan region (17, 18). In 2022, Costa Rica’s GDP *per capita* was $13,400, compared to the OECD average of $43,500 (19). The country has significant socioeconomic inequalities, with a Gini Index of 0.472, the second highest among OECD countries, surpassed only by Colombia (20). The wealthiest 10% of households receive 32% of net income, while the poorest 10% receive only 1.5% (17). There are also important differences between urban and rural areas. Urban areas have a 68% higher net income *per capita* compared to rural areas (21). Costa Rica is structured into three main geographical levels: provinces (7), cantons (81), and districts (489).

Despite the well-established relationship indicating that high GDP *per capita* lead to high life expectancy, Costa Ricans can expect to live up to 81 years (22), quite similar to high-income countries with higher GDPs (23). The country’s good vital statistics can be partially attributed to the national healthcare insurance system, which is constitutionally universal, mandatory, and based on citizen solidarity. Administered by the *Caja Costarricense del Seguro Social* (CCSS) since 1941, it covers an estimated 90% of the population (24). Funded by employees, employers, and the government, the CCSS established primary healthcare facilities (*Equipos Básicos de Atención Integral en Salud*—EBAIS) in the 1990s to decentralize healthcare to cover rural and underserved populations (25). Costa Rica has over 1,000 EBAIS, distributed throughout the national territory, each serving about 4,500 individuals with a team that includes a physician, nursing assistant, and primary care technical assistant. These teams are supported by shared staff, including social workers, nurses, nutritionists, pharmacists, microbiologists, and medical records technicians (26). The CCSS also manages 29 hospitals: 3 national (located in the metropolitan area), 6 specialized, 8 regional, and 12 peripheral (27). However, the country has only 1.2 hospital beds per 1,000 inhabitants, compared to the OECD average of 4.3 (28). Unlike many healthcare systems, there are no out-of-pocket expenses in the CCSS, ensuring that medical care and medications are provided free of charge within the public system. Nonetheless, some healthcare services are inadequately covered, leading to reliance on private providers. As a result, about 21% of healthcare expenditures in Costa Rica are out-of-pocket, similar to the OECD average of 18% (28).

The present study is based on the social epidemiology perspective that explicitly investigates the social determinants of population distributions of health, disease, and wellbeing, considering that health inequalities are a biological expression of social inequality (29). We based our search according to the conceptual framework of the WHO Commission on the Social Determinants of Health (30) that defines the social determinants of health as the “conditions in which people are born, grow, work, live, and age, and the wider set of forces and systems shaping the conditions of daily life. These forces and systems include economic policies and systems, development agendas, social norms, social policies, and political systems” (1).

Research on health inequalities in Costa Rica remains limited, lacking a comprehensive analysis of existing studies. The evolving political and economic circumstances, increasing urbanization, rapid population aging, and shifts towards more Westernized lifestyles underscore the need to report and monitor these disparities, as these circumstances can increase social inequality and thus become health inequalities. This is central to develop better-adapted policies and improving preventive health services.

In the present study, we assess health inequalities in Costa Rica, by reviewing existent literature on health disparities arising from social determinants, evaluating the impact of “equity stratifiers” on health status (31), such as, educational level, urbanicity, income, wealth and other social determinants of health, such as, access to healthcare and health behaviors. This systematic review seeks to provide a comprehensive understanding of the state of health inequalities research and contribute to the broader discourse on the role of social determinants of health in the country. Here, the terms “graded relationship” or “social gradient” refer to the observed phenomenon wherein individuals situated at lower socioeconomic strata experience poorer health outcomes compared to those at higher socioeconomic levels. A reverse social gradient occurs when individuals in lower socioeconomic strata experience better health outcomes compared to those in higher socioeconomic levels.

## 2 Methods

### 2.1 Search strategy

**Figure 1:**
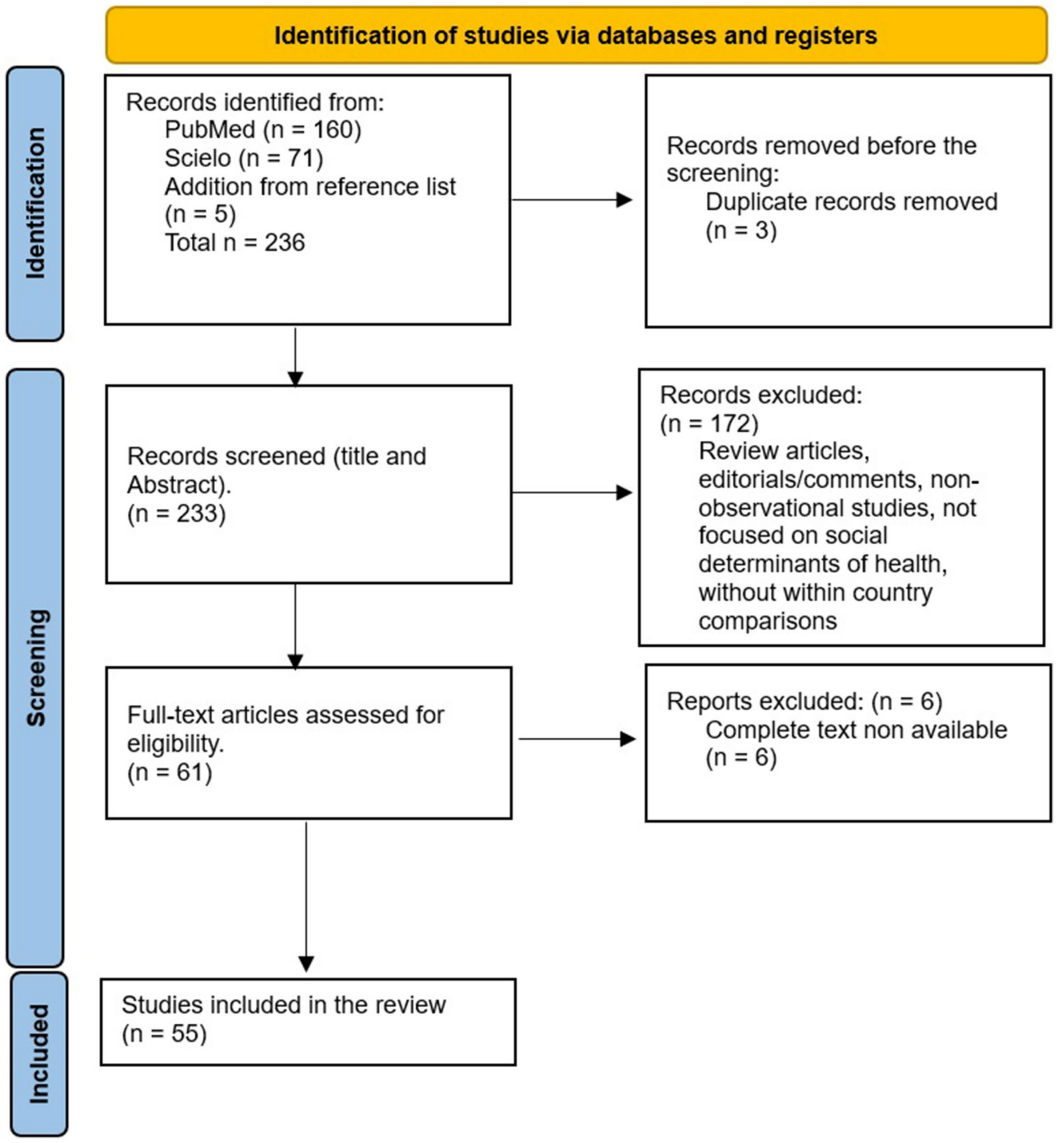
PRISMA flow diagram of the study. From: Mother et al. (32).

Articles assessing social inequalities in health in Costa Rica were retrieved. Published studies were identified by a systematic review in the PubMed electronic database, to identify English articles, and the Scielo electronic database for Spanish articles. PRISMA (Preferred Reporting Items for Systematic Reviews and Meta-Analyses) was followed. Flow chart for selection of articles is shown in Figure 1. Based on the PICO model, our approach involves: population (Costa Rican inhabitants), Intervention (considering social determinants as exposures), comparison (different socioeconomic subgroups), outcome (health inequalities in mortality, life expectancy, and morbidity). The PICO methodology was employed to select the main groups of search terms. For participants and location, we focused on observational studies conducted among Costa Rican inhabitants. For exposure, we included social determinants and socioeconomic status. For the outcome, we targeted health inequalities, such as inequality, inequity, disparity, and social gradient.

Article search was performed from December 2023 to January 2024 in PubMed and Scielo electronic databases. No publication date restrictions were imposed. Articles in English and Spanish were considered. One author (CBS) performed the electronic search in both databases and examined the titles and abstracts identified in the databases search, removed articles that did not meet the inclusion criteria and selected the eligible ones for full-text review. Additional articles (*n* = 5) were incorporated into the main reference lists that were not initially captured by the electronic search but were deemed relevant. Among these additions was a health agency report on drug consumption. However, we did not find other items of grey literature to include.

The following algorithm built with MeSH terms was used for the PubMed search: (“Costa Rica”[Title/Abstract]) AND ((“social determinant*”[Title/Abstract]) OR (“socioeconomic status”[Title/Abstract]) OR (“social gradient”[Title/Abstract]) OR (inequalit*[Title/Abstract]) OR (disparit*[Title/Abstract]) OR (inequit*[Title/Abstract])). For Scielo, the following algorithm was used: ((ab:(“Costa Rica”)) OR (ti:(“Costa Rica”))) AND ((ab:(“inequidades”)) OR (ti:(“inequidades”)) OR (ab:(“inequidad”)) OR (ti:(“inequidad”)) OR (ab:(“desigualdad”)) OR (ti:(“desigualdad”)) OR (ab:(“disparidad”)) OR (ti:(“disparidad”)) OR (ab:(“disparidades”)) OR (ti:(“disparidades”)) OR (ab:(“desigualdades”)) OR (ti:(“desigualdades”))).

### 2.2 Selection strategy

The eligibility criteria encompassed observational studies (including cross-sectional, case–control, and cohort studies) as well as ecological studies that reported on health inequalities in mortality, morbidity, health behaviors, risk factors, or those investigating the role of social determinants of health. These studies were required to be conducted in Costa Rica and to include within-country comparisons, with no limitation on publication date or language. Additionally, studies were expected to employ random sampling methods and be representative of at least one locality, city, or region within Costa Rica.

The exclusion criteria were the following: systematic reviews, literature reviews, commentaries, editorials. Studies whose objectives were not related to health outcomes were removed, as well as those articles that only made cross-country comparisons.

### 2.3 Data extraction

The article selection process involved two main steps. Initially, information on titles and abstracts from the entire set of results obtained from PubMed and Scielo electronic platforms was extracted and processed using a CSV format. Titles and abstracts of all references identified in the search were screened applying exclusion and inclusion criteria. Following this, the preselected articles underwent full-length article readings to identify and exclude any additional articles that did not meet the inclusion criteria.

For the studies finally selected, the following data were extracted: title, last name of first author, study objective, study design (including the cohort or study name), study period, sample size, characteristics of participants (including study period), social determinant used (exposure), health outcome(s) (outcome) and main results.

## 3 Results

This study is divided in four sections. The first section will lay out the main tenets of health inequalities in overall mortality (in general population, infants and older adults) and life expectancy, the second section will focus on cause-specific mortality. The third section will discuss the main determinants of health inequalities identified or risk factors that can mediate the link between the social environment and health. Finally, the findings and remaining challenges will be discussed.

The initial search strategy identified 232 articles (160 from PubMed and 71 from Scielo), and 5 papers were retrieved from reference lists that were not found in the search. The article selection process and flow chart are presented in Figure 1. Among these, three articles were found to be duplicated. After Title/Abstract screening, 172 articles were rejected for reasons such as only running cross-country comparisons, using Costa Rica only as an example for Latin America, being non-original studies (editorials, commentaries), or not analyzing associations between social determinants and health. Additionally, six articles were excluded due to the unavailability of the complete text. Finally, 55 articles were comprehensively evaluated.

### 3.1 General characteristics of the studies

General characteristics of the articles included in this systematic review are presented in Table 1. From the 55 articles 31 were cross-sectional, 20 longitudinal, 3 case–control and 1 qualitative study. Studies were conducted between 1984 and 2023 across various population groups. These groups include the general population (ages 20 to 89 years), infants (under 5 years), and older adults (60 years and above) (see Table 1) (33). Sixteen studies used ecological approaches, at the level of the province (7 provinces), county (~ 80 depending on the year in which the study was developed) or district (~ 478). Ecological studies extracted census data from 1984, 2000, 2011 (34), approached SES via household income and wealth (using material deprivation counting assets and characteristics of homes) and urbanicity (territories characterized as rural, mixed or urban). Studies using SES individual measures, collected data on income, education level (primary, secondary and more), occupation or subjective economic situation. They used cross-sectional data of specific projects: SALUBRAL, Global Adults Tobacco Survey, National School Weight/Height Census, Maternal and child National surveys (see Table 1) or were based on the Costa Rican Longevity and Healthy Aging Study (CRELES), including a representative sample of people born between 1945–1955, and a second cohort including people born before 1945 of about 8,000 adults (35).

**Table 1:** General characteristics of the studies included in the systematic review.

Study	Objective	Survey period	Study design	Study population	Sample size	Social determinant	Outcome	Mean age, range or distribution (%)	Male/Female
1. Assari and Lankarani (2015)	To compare 15 countries for the mediating effect of multi‑morbidity (chronic medical conditions) on the effects of demographic and socioeconomic characteristics on self-rated health.	NI	Cross-sectional study using the Costa Rican Study of Longevity and Healthy Aging cohort (CRELES)	Elders (age > 60 years)	*n* = 2,374	Income Education Gender Marital status	SRH	78.94 yo	NI
2. Barboza Solis and Fantin (2017)	To evaluate health inequalities in tooth loss, using indicators of socioeconomic Position.	2005	Cross-sectional using CRELES	Elders (age > 60 years)	*n* = 2,705	Education Occupation Subjective economic situation	Tooth loss	60 y and over	F: 54.3% M: 45.7%
3. Barboza Solis et al. (2021)	To identify socioeconomic inequalities in lip, oral cavity and pharynx (LOP) cancers incidence and mortality in Costa Rica.	Incidence: January 1, 2011, to December 31, 2015. Mortality: January 1, 2010, to December 31, 2018 Census data: 2011	Cohort study Incidence: from national population-based Cancer Registry Mortality: from National Death Index	Costa Ricans 20y and older	Incidence population: 2,798,517 with 601 new LOP cancers included. Mortality population: 2,739,733 586 cancer death included	Urbanicity Wealth from census data	LOP cancer Incidence: 601 cases LOP Mortality: 586	20y and older	Incidence: F: 238, M: 363 Mortality: F: 155, M:431
4. Barboza Solis et al. (2024)	To describe the geographical distribution of dental health practitioners according to urbanicity and area-level socio-economic status in Costa Rica	Dentist’s survey: 2021 Census data: 2011	Ecological study using GIS technologies Representative cross-sectional data from a dentist’s survey	Dentists and dental clinics	Dentists *n* = 3,810 Clinics *n* = 2,853	Urbanicity Wealth	Territorial distribution of dentists and clinics	20–39: 46% 40–59: 38% +60: 16%	F:35% M:65%
5. Bilal et al. (2019)	To examine inequalities in life expectancy in six large Latin American cities and its association with a measure of area-level socioeconomic status	Between 2011–2015 Mortality comes from Registry data between 2011–2015 SES measures comes from the 2011 National Census	Cross-sectional SALUBRAL Ecological study	San José inhabitants	2,500,000 followed	Sex/gender Area level socioeconomic status	Life expectancy at birth	NI	NI
6. Brenes-Camacho and Rosero-Bixby (2009)	To assess the level of preventive service utilization among Elders	2004–2006	Cross-sectional using CRELES first wave	Elders (age > 60 years)	n = 2,827	Education income	Use of social security prevention services	60y and over	
7. Carvajal and Geithman (1986)	To identify socioeconomic variables related to fertility	1963	Cross-sectional from Population Housing Census	Costa Rican households	n = 15,924 households	Household income	Number of children aged 0 to 10	Women between 15 and 55yo, children and spouse	NA
8. Chamizo García and Behm (2014)	To assess geographical inequities and social determinants in infant mortality	2008–2012	Cross-sectional Ecological study using Registry mortality data Cantonal data from the Human Development Index Census data for the environmental quality	Infants under 1 yo included	NI	Cantonal Human Development Index Cantonal Gini index Environmental Health Quality Index.	Infant mortality rate	From birth and 11 months	NI
9. Chamizo-García (2016)	To assess geographical inequities and social determinants in tuberculosis mortality and morbidity	2008–2012	Cross-sectional Ecological study using Registry mortality data Cantonal data from the Human Development Index Census data for the environmental quality	Individuals who had a diagnosis or died of tuberculosis	NI	Cantonal Human Development Index Cantonal Gini index Environmental Health Quality Index.	Mortality from tuberculosis	NI	NI
10. Chamizo-García (2016)	To assess geographical inequities and social determinants in diarrhea mortality	2008–2012	Cross-sectional Ecological study using Registry mortality data Census data for the environmental quality	Individuals who died of diarrheal diseases	NI	Cantonal Gini index Environmental Health Quality Index.	Mortality from diarrheal diseases	0—75y and over	NI
11. Chamizo-García (2021)	To analyze health inequities associated with conditions of environmental sanitation and access to health insurance	2018	Cross-sectional using the 2018 national household survey of Costa Rica	NI	NI	Education level, household poverty level	Household material deprivation: access to water supply, having a toilet, inadequate disposal of solid waste, cooking with firewood or charcoal, access to health insurance	Individuals aged 15y and over	NI
12. Espinoza-Aguirre et al. (2020)	To determine the association between sociodemographic characteristics and the prevalence of current tobacco consumption in Costa Rica	2015	Cross-sectional using data from the Global Adults Tobacco Survey (GATS).	Representative study of Costa Ricans 15y and older	n = 8,607	Urbanicity Education Household wealth, Household composition	Current smoking status	15 years and over	F: 50.3% M: 49.7%
13. Fantin et al. (2018)	To explore the role of early socioeconomic circumstances on tooth loss, using a life course mode	2010	Retrospective cohort study using CRELES 1945–1955 Retirement Cohort	Nationally representative longitudinal survey of Costa Rican residents born between 1945 and 1955	n = 2,796	Early socioeconomic conditions, Current socioeconomic conditions, Education Urbanicity	Adult tooth loss	Mean age: 59.7 (SD 3.2)	F: 61% M:39%
14. Fantin and Barboza (2020)	To analyze the association between cancer incidence and socioeconomic position in CR	January 1, 2011 to December 31, 2015	Cohort study using Costa Rican Cancer Registry, a national population-based registry followed. SES measured at ecological level	From the National Electoral Rolls were included all Costa Rican citizens aged 18y and older	Population followed: 2,798,517 *n* = 44,799 new cancer cases	Urbanicity Wealth by estimating the percentage of people with at least one Basic Unmet Need (BUN) from census data	Cancer incidence	Individuals aged 18y and over	F: 50.1% M: 49.9%
15. Fantin and Barboza (2020)	To analyze the relationship between the socioeconomic characteristics of patients’ residential districts and mortality due to cancer in CR	January 1, 2011 to December 31, 2017	Cross-sectional Ecological study at the level of the district of residence using the Death Index Registry.	All cancer-caused deaths	Population: 3,740,654 *n* = 32,117 cancer mortality cases included	Urbanicity Wealth by estimating the percentage of people with at least one Basic Unmet Need (BUN) from census data	Cancer mortality	NI	F:48.9% M: 51.1%
16. Fantin and Barboza (2020)	To analyze 5-year net survival after cancer diagnosis CR, according to the characteristics at the district level	January 1, 2011 to December 31, 2015	Cohort study using Costa Rican Cancer Registry, a national population-based registry. Deaths occurring before December 31, 2018 were identified using the National Death Registry. SES measured at ecological level.	Costa Rican inhabitants with a cancer diagnosis	n = 46,904 cancer cases included	Urbanicity Wealth by estimating the percentage of people with at least one Basic Unmet Need (BUN) from census data	Cancer survival (5-year survival)	15 and 99 yo	F: 55% M: 45%
17. Fantin and Barboza (2020)	To determine the existence of life expectancy inequities according to province of birth and sex in CR	2013–2017	Cohort study using the Birth and Death Index Registry	Costa Rican inhabitants who died between 2013 and 2017	n = 93,298 death included	Birth province	Life expectancy at birth	0 to 100 yo	F: 49.6% M: 50.4%
18. Fantin et al. (2021)	To assess the association between the socioeconomic characteristics of the place of residence and life expectancy in CR	January 1, 2010 to December 31, 2018	Cohort study using the National Death index and National Electoral Rolls. SES measured at ecological level	Adult Costa Rican citizens	2,747,616 people followed for *n* = 23,985,602 person-years of follow-up	Urbanicity Wealth	Life expectancy	18 y and over	F: 50.1% M: 49.9%
19. Fantin and Barboza-Solis (2022)	To analyze the differences in mortality and causes of death between people living in indigenous areas, and people living in the rest of CR	January 1, 2010 to December 31, 2018	Cohort study using the National Death index and National Electoral Rolls.	Adult Costa Rican citizens	2,747,616 people followed for *n* = 23,985,602 person-years of follow-up were included	Indigenous zones	Mortality rate	18 y and over	NI
20. Fantin et al. (2023)	To analyze health inequalities in cause-specific mortality in CR, observing the main causes for inequality in the country	2010 to 2018	Cohort study using the National Death index and National Electoral Rolls. SES measured at ecological level.	Adult Costa Rican citizens	Population 2,739,733 *n* = 153,815 death causes included	Urbanicity Wealth	Cause-specific mortality	20 y and over	F: 50.1% M: 49.9%
21. Fantin et al. (2023)	To analyze the mortality associated with the COVID-19 pandemic in CR	2017–2021	Cross-sectional	Costa Ricans	7,640 deaths attributed to COVID-19 between March 2020 and December 2021	Urbanicity District wealth	Mortality from COVID-19	0–100 yo	F:49.6% M: 50.4%
22. Fantin (2024) Accepted in Cancer Epidemiology	To analyze the SES inequalities in cancer mortality in CR	2010–2018	Cohort study using the National Death index and National Electoral Rolls. SES measured at ecological level.	Costa Ricans	Deaths from cancer *n* = 38,656	Urbanicity Wealth	Cancer mortality	20 years and older	F: 50.0% M: 50.0%
23. Gamboa-Gamboa et al. (2021)	To analyses the relationship between SES and the prevalence of overweight and obesity in the primary school population in CR	2016	Cross-sectional using a National School Weight/Height Census	Students from 6 to 12 years enrolled in public and private primary schools.	347,366	Type of institution (private, public, mix); Urbanicity Level of development of the district of residence (in quartiles)	Overweight and obesity	6 to 12yo	M: 51.4% F:48.6%
24. García-Marín and García (2022)	To assess the relationship between the transmission gross rates and death rates from COVID-19 and with socioeconomic and health indicators	June 2020 to March 2021	Cohort study	Costa Rica inhabitants	NI	Cantonal indexes: Social Progress Index Human development Index	COVID-19 infection and death	NI	NI
25. Goldman et al. (2011)	To assessed the extent to which biological markers of chronic disease account for social disparities in health	2004–2006	Cross-sectional analysis using the first wave of the Costa Rican Study on Longevity and Healthy Aging (CRELES)	Nationally representative sample of adults born in 1945 or earlier	n = 2,827	Educational attainment	SRH Functional limitations Biomarkers	60 and over	NI
26. Gómez (2021)	To investigate the impact of SES on diet quality and body mass index in Latin America	September 2014 to June 2015	Cross-sectional from the “Latin American Health and Nutrition Study (ELANS)”	Representative of Costa Ricans 15-65y	n for CR = 798	Head of house’s income, occupation education Persons living in the house, House ownership, house area, type of construction, number of rooms, having water, gas, electricity supply Personal assets (e.g., vehicles, computers)	Dietary intake Diet quality Diet diversity Nutrient Adequacy Weight Status	15–65 years	NI for CR
27. Herrera-Cuenca et al. (2021)	To determine the prevalence of obesity and stunting in the Latin American population living in the eight countries	2014–2015	Household-based multicenter cross-sectional Study from the “Latin American Health and Nutrition Study (ELANS)”	Representative of Costa Ricans 15-65y	n = 551	SES (no variables indicated)	Obesity Waist circumference Neck circumference Stunting Waist-hip ratio Calcium intake Iron intake	15–65 years	NI
28. Herrero et al. (1993)	To study geographic variation of invasive cervical cancer in CR	1986–1987	Case—Control	Women with cervical cancer	Cases *n* = 192 Controls *n* = 372	Residence Education level SES (Household utilities)	Cervical cancer	NI	NA
29. Hong et al. (2016)	To explore sociogeographic inequalities in the availability and distribution of ophthalmologists across 14 Latin American countries	2013	Cross-sectional Ecological study comparing the availability of ophthalmologists per population in capital areas vis-à-vis of the rest of the country	NI	NI	Regional Human Development Index Inequality concentration index Index of dissimilarity	Mean level of ophthalmologists per population	NI	NI
30. Hyland et al. (2018)	To examined whether self-reported maternal pesticide use inside the home and nearby pesticide applications before and after the child’s birth were associated with acute lymphoblastic leukemia	2001–2003	Case—Control	Children diagnosed with acute lymphoblastic leukemia	Cases *n* = 251 Controls *n* = 577	Parental education SES	Acute lymphoblastic leukemia	Cases: <1y: 4% 1y-4y: 45.8% 5y-9y: 33.1 10y-15y: 17.1%	Boys (cases): 54.6% Boys (controls): 49.0%
31. IAFA (2018)	To document drug consumption in the general population	2015	Cross-sectional	Costa Rican population	n = 15,876	Urbanicity	Drug consumption	12–70 yo	NI
32. Llorca Castro and Ortún (2010)	To analyze the variations of the unnecessarily premature and sanitarily avoidable mortality of each of the 81 cantons of Costa Rica during 2000–2005.	2000–2005	Cross-sectional Ecologic study at the canton level	Costa Rican population between 2000–2005	32,126 deaths cases included	Indicator of Socioeconomic Development created by the University of Costa Rica	Absolute mortality	0–74 yo	NI
33. Lozoff et al. (2006)	To assess change in cognitive functioning following iron deficiency in infancy, depending on socio-economic status	1981–1984 to 2000–2002	Cohort study	Children enrolled at 12–23 months and followed at 5, 11–14, 15–18, and 19 years	Baseline *n* = 185 19y wave *n* = 121	Hollingshead Four Factor Index	Iron deficiency	19y	NI
34. Mazariegos et al. (2021)	To assess the association between obesity and education levels and explored the potential effect modification of this association by city-level socio-economic development	2005	Cross-sectional study from SALURBAL data	Individuals living in San José	n = 1,164	Education level	Obesity	Adults aged >18 years old	F: 63.7% M: 36.3%
35. Metzger (2002)	To compare how different degrees of data aggregation impact the results of measuring health inequalities	1973 and 1984	Cross-sectional using Registry data on infant mortality	Costa Rican infants	1973 *n* = 771 1984 *n* = 556	Different SES measures (Gini index, income, education from census data)	Infant mortality	NI	NI
36. Monge-Rojas et al. (2021)	To develop and validate a Traditional Costa Rican Adolescents Diet score and determine its sociodemographic correlates	2017	Cross-sectional	Adolescents in rural and urban schools in the province of San José	*n* = 804	Residence Parental education Goods ownership Access to services (e.g., computers, internet, water heating, etc.) Number of people in the household, Number of bathrooms	Dietary intake Traditional Costa Rican Adolescents Diet (TCRAD) Score	13–18 yo	Girls: 63.6% Boys: 36.4%
37. Modrek et al. (2012)	To evaluate the relationship between mortality and economic inequality in CR	January 1, 1989 to December 31, 2007	Cohort study using the Death Index Registry and the Costa Rican Longitudinal Mortality Study. SES measured at ecological level.	Costa Rican adults	n ∼ 16,000	Canton-level income inequality measured using Gini coefficients, Relative deprivation based on asset ownership	Time to death Cardiovascular disease mortality.	Adults 30 and over	NI
38. Mujica et al. (2023)	To assess trends in maternal and child health inequalities in Latin America and the Caribbean	2011 and 2018	Cross-sectional	National survey of women and children	*n* = 5,084 women *n* = 2,272 children	Household wealth indices based on assets and characteristics of the homes: (Q1: poorest, Q5: richest)	Composite coverage index, demand for family planning, antenatal care, skilled attendant at birth, postnatal care, full immunization coverage, stunting among under-five children, tobacco use	15-49yo and children	NA
39. Núñez-Rivas et al. (2020)	To analyze the diet of students in Costa Rica with construction of a contextualized new diet quality index	NI	Cross-sectional	Children and adolescents in school	n = 2,677	SES (low, middle, high)	Diet Quality Index	7–18 yo	F: 43% M:57%
40. Poirier et al. (2022)	To explore socioecological wellbeing among Nicaraguan migrant workers in Costa Rica	2021	Qualitative methods	Nicaraguan migrant workers	13 in-depth interviews and 2 indepth interviews with an expert and an activist	Structural discrimination	Sociological wellbeing	NI	NI
41. Restrepo-Mendez et al. (2015)	To describe SES inequalities in maternal health coverage, mortality, stunting.	CR: 2011	Cross-sectional	Nationally representative study of women and children	NI for CR	Household wealth indices based on assets Characteristics of the homes: (Q1: poorest, Q5: richest)	Composite coverage index	NI	NI
42. Rosero-Bixby (1991)	To identify the main factors (health interventions and social development) explaining the mortality decline in CR	1940–1980	Cross-sectional data from mortality registry	Costa Ricans	NI	Health interventions	Mortality rate	NI	NI
43. Rosero-Bixby (2004)	To analyze a geographic information system (GIS) to relate the population’s demand with an inventory of health facilities (supply)	2000	Cross-sectional Ecological study	Costa Rican’s Public health facilities	NA	Distance to public medical facilities	Territorial distribution of public health facilities and access to health care	NA	NA
44. Rosero-Bixby (2005)	To report on the relationship between health insurance and longevity	1984–2001	Cohort study	Prospective panel study of about Costa Rican adults	n = 876	Education (completed elementary school or not) Household wealth (number of assets)	Mortality rate	60 and above	F: 53% M: 47%
45. Rosero-Bixby (2009)	To determine SES gradients in different dimensions of health among Elders Costa Ricans.	2000–2007	Cohort CRELES	Costa Rican Elders	n = 8,000	Education Income Wealth	Mortality, Metabolic syndrome, frailty, depression, SRH, biomarkers,	60y and over	F: 53% M: 47%
46. Rosero-Bixby (2016)	To compare the SES gradients in adult mortality and health risk factors in Costa Rica with comparable data in the United States	USA: 1992–1998 CR: 1990–2002	Comparative Cohort study study of two datasets: The US National Health and Nutrition Examination Survey (NHANES) and NLMS-CR	USA population Costa Rican population	USA *n* = 288,000 Number of deaths: 22,440 CR *n* = 17,500 Number of deaths: 2,415	Education Household wealth Family support, Insurance status Smoking BMI Sedentariness Biomarkers	Mortality risk	40–89 yo	NI
47. Rosero-Bixby (2018)	To estimate mortality at older ages in two Latin American countries (Mexico and Costa Rica) using recent longitudinal surveys and to determine the SES gradients	Mexico 2001–2012 CR: 2000–2014	Cohort study	Mexico *n* = 6,700 CR *n* = 7,200		Educational attainment (no formal education, some elementary, some secondary, and postsecondary education); Household wealth (household assets) Urbanicity	Life expectancy at 60y	55–99 yo	NI
48. Santamaría-García et al. (2023)	To investigate the combined impact of social determinants of health, lifestyle, cardiometabolic factors, mental health and demographics on healthy aging across LAC countries	2012–2016	Cohort study including two waves of CRELES	Costa Rican adults	n = 5,694 original sample With complete data *n* = 1751	Education level	Cognition and functional ability	Mean: 59.5yo	NI
49. Santamaría-Ulloa et al. (2019)	To quantify cervical cancer incidence inequality along three decades and to explore its determinants	2000–2010	Cohort study Ecological study using National Cancer Registry and the CRELES cohort study	Costa Rican women	n = 21,075 incident cases included	Inequality index: Theil-T index, economic condition, access to healthcare and sub-utilization of Pap screening.	Cervical cancer incidence	15y and over	NA
50. Santamaría-Ulloa et al. (2019)	To estimate inequalities in diabetes incidence, prevalence and mortality and assess the economic burden on the healthcare system in CR	2004–2009	Cohort study (CRELES)	Costa Rican adults	n = 2,827	Income, education level	Mortality, Diabetes status	60y and over	NI
51. Santamaría-Ulloa and Bekelman (2021)	To examine the association between processed meats intake and total protein intake, inadequate protein intake, by SES	2014–2015	Cross-sectional	Women living in two counties from the Greater Metropolitan Area of San José	n = 135	SES index including: having completed secondary school, employment in the formal sector, home located on a paved street, housing with a polished wooden or ceramic floor, neighborhood located in a canton with a high Human Development Index.	Inadequate protein intake (calculated from the daily protein requirement per person), processed meats intake	25-yo	NA
52. Santamaría-Ulloa et al. (2022)	To determine if there are differences in the use of Pap smears at the regional level in CR, to observe inequalities related to the early detection of cervical-uterine cancer	2014 using the National Household Survey	Cross-sectional	Women living in CR	n = 11,578	Geographical regions (residence), Education level	Pap screnning: time since the last time had a Pap smear, reasons why women had never had a Pap smear	Women from 20 to 64 years old	NA
53. Sotos-Prieto et al. (2016)	To assess the associations between two cardiovascular risk scores (lifestyles and genetic) on myocardial infarction	1994 and 2004	Case—Control	Costa Rican adults	n = 1,534	Socioeconomic score (quantitative variable) using education, occupation, household income, and household assets Urbanicity	Myocardial infarction	Controls: 57.9 (11.1) Cases: 58.1 (10.9)	F (cases): 24.6% F (controls): 24.6%
54. Sudharsanan et al. (2020)	To implement an international-comparative study on schooling differences in adult mortality across several MICs using nationally representative data	NI	Cohort using CRELES	Costa Rican Elders	*N* = 2,603 number of deaths: 504	Education level (no completed, primary, secondary, and tertiary schooling)	Mortality risk	65 and over Median: 75yo	F: 54% M: 46%
55. Valdivieso et al. (2022)	To evaluate the measurement invariance of a neuropsychological battery across rural and urban older adults from Costa Rica	2014–2016	Cross-sectional from the Epidemiology and Development of Alzheimer′s Disease (EDAD) study in Costa Rica	Rural and urban older adults	*n* = 295	Urbanicity, Education leve	Measurement invariance of neuropsychological batteries (cognitive abilities)	60–85 yo	F: 76.9% M: 23.1%

NA: Not applicable. NI: Not Indicated. CR: Costa Rica. CRELES: Costa Rican Study of Longevity and Healthy Aging cohort. NLMS: Longitudinal Mortality Study of Costa Rican Adults. LAC: Latin American and the Caribbean. Y: Years. Yo: Years old. MICs: Middle-Income country.

To assess health outcomes, the authors of the studies included in this review utilized the Costa Rican registries, including birth, mortality, and cancer registries, which have been described as comprehensive (36). The cancer registry is considered 100% complete, which is an exceptional country effort (37). Using data from the national cancer registry, several studies characterized the social distributions in cancer incidence, mortality and survival. A Longitudinal Mortality Study of Costa Rican Adults (NLMS) 1984–2007 merged data from the Death Index Registry and the 1984 Census, following more than 16,000 people aged more than 30 years between January 1, 1989 and December 31, 2007, and observed more than 3,700 deaths. Studies also included outcomes from specific studies (e.g., anthropometric measures, cardiovascular health, Diabetes, maternal and child health, self-rated health), collected by cross-sectional studies or from the CRELES cohort. A literature review article (not included in this analysis) presented the main methodologies used to analyze health inequalities in Costa Rica (38).

### 3.2 Health inequalities in mortality and life-expectancy

#### 3.2.1 In general population

The most extensive study examining mortality inequalities through individual socioeconomic data was conducted by Rosero-Bixby and Dow (39), utilizing data from the Costa Rican Longevity and Healthy Aging Study (CRELES) and the National Longitudinal Mortality Study (NLMS). Their findings revealed a higher mortality rate among individuals in the lowest (SES) ranks compared to those in the highest SES, consistent with results reported by Modrek et al. (40), who used only NLMS data. Rosero-Bixby and Dow (39) found that higher SES was associated with lower mortality rates in the 1990s, indicating a positive social gradient in health (RII = 1.3, CI [1.1–1.5]). The authors observed that mortality decreased with increasing education and wealth. Rural residents exhibit lower mortality rates than their urban counterparts after adjusting for wealth and education. Stratification by age groups revealed social gradients among men and women aged 40–64 years (Relative Inequality Index (RII), RII = 1.3 in men, RII = 1.7 in women), as well as among women aged 65–89 years (RII = 1.5) (39), but no significant gradient was observed among men in the 65–89 age group (RII = 0.8, non-significant). Interestingly, these social gradients were notably less pronounced than those observed in the United States (RII = 3.5 in men, RII = 3.8 in women).

These results are consistent with a recent study conducted by Fantin et al., which followed all citizens alive in 2010 (24-million person-years follow-up) between 2010 and 2018 (41). The study utilized the district level as the smallest administrative unit to assess SES of each individual. Among both men and women residing in urban areas, those living in the wealthiest districts exhibited a life expectancy more than 2 years higher than those in the poorest districts. Similar trends, but with less pronounced disparities, were observed in rural areas. However, among men, the protective effect of residing in rural areas resulted in comparable mortality rates between the poorest districts (predominantly rural) and the wealthiest districts (mainly urban).

In studies using larger geographical units, such as the canton level, associations between area-level SES and life expectancy become less apparent. For instance, Bilal et al. (12) described geographical disparities but did not find a significant link between ecological socioeconomic level and life expectancy. Similarly, Fantin et al. (42) found relatively similar life expectancies among women when using the province at birth level. However, among men, there was a notable difference in life expectancy, with those born in Limón experiencing over 2 years of lower life expectancy compared to those born in almost all other provinces. The homicide rate and mortality from traffic accidents in Limón partially explain the findings; however, the association continues to be significant after adjustment.

#### 3.2.2 Specific population: infants

In 2021, Costa Rica exhibited low infant and under-5 mortality rates, standing at 6 and 8 per 1,000 live births, respectively (43). Consequently, there are few recent studies, especially on social inequalities. Chamizo-García and Behm-Ammazzini (44) suggested that infant mortality between 2008 and 2012 was slightly higher in the poorest cantons, particularly those located in the southern region of the capital, border areas, or coastal regions. These findings align with previous results from the 1960s and 1980s, during which infant and under-5 mortality rates were significantly higher (45, 46). In 1991, Rosero-Bixby proposed that from 1965 to 1979, infant mortality was closely linked to maternal education level, whereas between 1981 and 1984, the association between infant mortality and maternal education level became less clear (46). Metzger (47) confirmed these results, showing a strong social gradient in 1973, that disappeared in 1984.

#### 3.2.3 Specific population: older adults

In both 2009 and 2016, Rosero-Bixby and Dow observed no social gradient in mortality within the CRELES cohort. Interestingly, they even noted a small reverse gradient among men, employing education level as a SES measure (48). In a separate study, Rosero-Bixby reaffirmed his previous findings, indicating comparable life expectancy at age 60 across different wealth levels (49). Using the education level as proxy of SES, the study showed a small social gradient in women, and a clear reverse social gradient in men. Finally, in men only, life expectancy was higher in rural area compared to urban area. Focusing on a 2,700-participants subsets of the CRELES cohort aged 65 years and above, Sudharsanan et al. (50) found no evidence of a social gradient in mortality according to education level but the follow-up and the sample size were lower compared to Rosero-Bixby’s analysis.

Two additional studies using data other than CRELES, analyzed mortality in older adults. Following a cohort of 876 individuals aged 60 and over between 1984 and 2001, residents of a semi-urban community near the capital (Coronado canton), Rosero-Bixby showed a protective role of education, but no effect of wealth on mortality (51). Finally, Fantin et al. observed that among individuals aged 60 years and older, there was an association between district wealth and mortality in both men and women, evident only in urban areas and not in rural areas. These findings do not necessarily conflict with those of CRELES, as the latter study did not stratify its results by urbanicity. Moreover, Fantin et al. noted that socioeconomic inequalities based on district wealth were less pronounced among individuals aged 60 years and older compared to those aged 20 years and older (41). Consequently, among men aged 60 years and older, life expectancy was 0.7 years higher in the poorest cantons of rural areas compared to the wealthiest cantons of urban areas.

### 3.3 Health inequalities in mortality, by cause of death

#### 3.3.1 Diseases of the circulatory system

Between 2013 and 2022, diseases of the circulatory system represented 28% of the deaths in women and 25% in men in Costa Rica (52). Various studies converged to show a social gradient in circulatory system mortality. In the 1990s, Rosero-Bixby and Dow (39) identified a social gradient in mortality associated with cerebrovascular disease (RII = 1.5), but not in mortality associated with heart disease (39). Using the NLMS data, Modrek (40) showed lower mortality rates for cardiovascular disease among high-educated individuals, a finding consistent with the results reported by Fantin et al. (41) based on mortality data observed between 2010 and 2018. Indeed, Fantin et al. identified a slightly lower mortality rate in the wealthiest districts compared to the rest of the country, although this finding emerged only after adjusting for urbanicity. Consistently, in a case–control study, people who had a myocardial infarction were found to have, on average, a lower SES compared to people who did not have myocardial infarction (53).

#### 3.3.2 Cancer incidence and mortality

Between 2013 and 2022, cancers represented 24% of the deaths in women and 21% in men in Costa Rica (52). Stomach, colorectum, prostate, breast, and lung, represent half of the all-cancer mortality. In the 1990s, Rosero-Bixby and Dow (39) did not show a social gradient in cancer mortality. Fantin et al. (54) confirmed the absence of social gradient in cancer mortality, but observed a lower mortality in rural areas compared to urban areas, consistent with a previous study (55).

Fantin et al. (56) detailed this result by cancer site. The authors observed a social gradient in cancer mortality for stomach, lung and cervical cancers, consistently with previous results on cancer incidence in Costa Rica (57). Inversely, they observed a reverse social gradient for colorectal cancer, non-Hodgkin lymphoma and leukemia. A reverse social gradient had already been found in the incidence of colorectal cancer (57). A lower mortality in rural areas was observed for most cancers, especially those associated with smoking, but also cervical cancers, prostate and breast cancers (56). Using the same methodology, Barboza-Solis showed in 2021 (58), that people who lived in the most socioeconomically disadvantaged districts had lower probabilities of developing lip, oral cavity and pharynx cancers, than people in the richest districts. Moreover, no evidence was found of a difference according to urbanicity or rurality in lip, oral cavity and pharynx cancers.

Fantin’s mortality and incidence results on cervical cancers are consistent with previous studies. Herrero et al. documented that cervical cancer incidence was higher in low-socioeconomic status, and in coastal areas in the mid-80’s (59). More recently, Santamaría-Ulloa et al. (60) confirmed these results showing a higher incidence in coastal and border areas, where populations are usually less favored.

Fantin’s findings regarding mortality from leukemia are not consistent with the results reported by Hyland et al. (61), who observed no differences in education levels between cases and controls between 1995 and 2000. The differences between the studies might be due to the outcome (mortality versus incidence), the measure of socioeconomic position (district’s wealth versus individual measures) or the sample size (1,449 deaths against 252 cases).

Llorca and Ortún (62) analyzed cancer mortality according to canton financial conditions, and showed an association between breast, uterus and skin cancers mortality and better financial conditions; and an association between prostate benign hyperplasia and financial conditions. Skin cancer mortality has also been found to be higher in rural compared to urban areas, consistently with Fantin’s results on cancer incidence. Llorca found that breast cancer mortality is higher in urban area. Fantin’s results confirmed this association, but attributed it to wealth after adjustment.

#### 3.3.3 Diseases of the respiratory system

Between 2013 and 2022, diseases of the respiratory system represented 9% of the deaths in women and 8% in men in Costa Rica (52). In pre-COVID-19 studies, Rosero-Bixby and Dow (39) in the 1990s and Fantin et al. (41) between 2010 and 2018 found strong social gradients regarding mortality associated with diseases of the respiratory system. The findings of Rosero-Bixby and Dow showed a relative inequality index (RII) of 2.5, (CI [1.5–4.3]). Despite being a rare outcome, Chamizo-García showed that mortality associated with tuberculosis was higher in the poorest cantons compared to the richest cantons (63). The author also suggested that material deprivation is more frequent in extreme poverty households, and where the head of the household has elementary school (64). In relation to COVID-19, Fantin et al. (65) did not identify significant differences based on district wealth. However, the authors observed a lower excess death rate in rural areas compared to urban areas (11% versus 18%), indicating fewer additional deaths attributable to COVID-19 when compared to previous years (65). Using the canton geographical scale, García-Marín confirmed a higher COVID-19 infection risk in urban areas and in poorer areas, but did not find association with mortality risk (66).

#### 3.3.4 External causes of mortality

Between 2013 and 2022, external causes of mortality (mainly car accidents, homicides and suicides) represented 5% of the deaths in women and 15% in men in Costa Rica (52). Car accidents, homicides and suicides represent 5, 4 and 2% of the deaths in men respectively, and 1, 0.5 and 0.6% in women. Rosero-Bixby and Dow in the 1990s (39) and Fantin et al. between 2010 and 2018 (41) found strong social gradients regarding mortality associated with external cause of mortality (54). Fantin et al. observed a more pronounced social gradient in men compared to women. This finding corroborates the results observed in the Limón province, where the lower life expectancy was partly attributed to higher rates of car accidents and homicides (42).

#### 3.3.5 Other causes of death

In the study on cause-specific mortality between 2010 and 2018, Fantin et al. identified that the two main causes of deaths (diseases of the circulatory system and neoplasms) showed no or a small social gradient, but most of the other causes of deaths had a clear gradient, in particular for mortality associated with infectious diseases, endocrine, nutritional and metabolic diseases, and diseases of the genitourinary system (54). These social patterns increased after adjustment for urbanicity, that can be explained by a protective effect of living in rural area, in particular in men. The only cause of deaths showing a reverse social gradient were the diseases of the nervous system (54).

Chamizo-García showed an association between 2008–2013 mortality for diarrheas and canton development (67). In its analysis at the canton level, Llorca (62) also indicated that mortality for HIV infection was higher in the wealthiest cantons, and that maternal mortality was lower in wealthiest cantons compared to the poorest.

#### 3.3.6 Self-reported health

Using CRELES data in elders, Assari et al. (68) and Rosero-Bixby and Dow (48) found that self-rated health increases with education level and income. Rosero-Bixby and Dow (48) additionally observed that physical and functional disabilities were lower in high-SES compared to low-SES elders. In particular, people without elementary school were at higher risk of functional disability (OR = 1.18, people with elementary school being the reference) than people with high school education (OR = 0.42). Similar results were found for physical frailty (OR = 1.18 and 0.58, respectively).

#### 3.3.7 Depression

There is little information regarding mental health and SES. Rosero-Bixby and Dow (48) found higher prevalence of geriatric depression in low-wealth (OR = 1.51, medium-wealth being the reference) compared to high-wealth (OR = 0.73) using the CRELES data.

### 3.4 Health determinants

#### 3.4.1 Access to health care

The public health system in Costa Rica provides free access to healthcare for the majority of the population (26). However, in other countries, it has been shown that this does not guarantee equitable distribution of healthcare access across all segments of the population. In 2004, Rosero-Bixby highlighted that the conception and strengthening of the preventive health units (EBAIS) has been pivotal in improving equitable access to healthcare, particularly in rural and impoverished areas. The authors noted that half of Costa Ricans reside less than 1 km away from an outpatient care facility and within 5 km of a hospital. Nonetheless, approximately 12–14% of the population was classified as underserved (69).

Moreover, some people are uninsured, in particular migrants. According to the last national surveys, 10% of the population declared themselves uninsured (70). During the COVID-19 pandemic, a qualitative study conducted with migrants showed that despite the well-known social welfare policies, migrant workers face additional burdens of exclusion and financial barriers (71).

Fantin et al. showed that cancer survival rates post-diagnosis were positively correlated with the wealth of districts, implying potential challenges in accessing timely diagnoses in economically disadvantaged areas. Interestingly, cancer survival rates were found to be similar between rural and urban areas (72). Additionally, Santamaría-Ulloa et al. highlighted in a 2022 study (73) that women residing in economically disadvantaged coastal regions (Chorotega, Central Pacific, and Huetar Caribbean) were less likely to participate regularly in cervical cancer screening. Furthermore, the study found that education level served as a protective factor for undergoing Pap screening and was associated with the reasons explaining why some women did not undergo any Pap screenings.

Brenes-Camacho assessed in 2009 health inequalities in the utilization of preventive health care services in people 60 years and over (25). The group with lower education levels was less likely to maintain blood pressure screenings and controls, as well as to undergo prostate screenings, mammograms, and eye examinations. This finding confirms Santamaria-Ulloa’s results, which indicated better access to screenings among high-SES individuals (73). Contrary to Santamaria-Ulloa’s findings, low-SES individuals were more likely to have frequent Pap smears. However, this discrepancy can be explained by the fact that most participants in Brenes-Camacho’s study were older than 65 years and thus not targeted for Pap smear screenings. Nevertheless, Brenes-Camacho et al. found that low-SES individuals were more likely to receive vaccinations (influenza, tetanus). This result aligns with the observations of Mujica et al. and Restrepo-Méndez et al., who noted in the 2010s that immunization coverage was less prevalent among the wealthiest quintile (74, 75).

Regarding maternal health care, studies did not find a social gradient in health coverage (75). Mujica et al. observed in the 2010’s that women in the richest quintile (Q5) had more antenatal care, compared to, respectively, Q4, Q3, Q2 and Q1 (having the most restricted access). In a classic article from 1986, it was already manifest in 1963, that the number of children decreased with income (76).

When it comes to access to health care that is poorly provided by the public service, inequalities become manifest. Hong et al. (77) observed, using measures of relative inequality, a disproportionate concentration of ophthalmologists among the economically advantaged areas, mainly when comparing the capital versus the rest of the country. Hong et al. found an inequality concentration index of 0.46 in Costa Rica versus 0.26 in average in Latin America. Similarly, dental care is mainly supported by private dentists. Barboza-Solís et al. indicated that dentist’s density was far higher in the wealthiest urban communities, compared to the poorest and/or the rural areas. Local Potential Accessibility to dentists was 9.3 per 100.000 inhabitants in the wealthiest districts against 6.2 in the most disadvantaged districts in the urban area, and 5.2 in the wealthiest districts against 1.9 in the most disadvantaged districts in the rural area. As a result, 16% of the Costa Ricans had very low or no access to dental care (78).

#### 3.4.2 Alcohol consumption

In its report on alcohol consumption based on a national survey in 2015 (79), the Institute on Alcoholism and Drug Dependence (IAFA), indicated that alcohol consumption in the past twelve months was strongly associated with household income in men and in women. Consuming alcohol was 68% in men and 54% in women among the wealthiest households, compared to 33% in men and 19% in women among the poorest households.

This is consistent with Llorca and Ortún’s findings, which showed that mortality due to hepatic disease (secondary to alcohol consumption) was higher in the wealthiest cantons compared to the poorest cantons (62).

#### 3.4.3 Smoking

Smoking prevalence sharply decreased in the last decades. Espinoza-Aguirre et al. (80) estimated smoking prevalence in 2015 to be 13% in men and 4% in women, using a representative sample of Costa Ricans. People who live in rural areas smoke less than those living in urban sites (7% vs. 10%). In 15–34 years age group, smoking prevalence was higher among individuals with low SES, but there was no association between education level or income and smoking prevalence in adults older than 35 years old. The absence of clear social gradient according to the household income in men was confirmed in a IAFA survey which used the household income in 2015 (79). In women, the results of the different surveys have contradictory results on social gradient, but all pointed to the low smoking prevalence (79, 80).

#### 3.4.4 Chronic diseases

All the studies presented in this section used the CRELES data and are therefore focused on elders.

Santamaría-Ulloa et al. presented a lower diabetes prevalence in elder population who completed primary school compared to those who did not, but the authors did not show association between diabetes prevalence and income (81). Longer time to the closest facility translates into a lower probability of having the condition diagnosed, which can indicate that diabetes prevalence is lower or that being diagnosed is more difficult in rural areas (81).

Rosero-Bixby and Dow (48) showed no difference in the prevalence of uncontrolled dysglycemia according to SES, but found a higher prevalence of uncontrolled hypertension in low-SES individuals. In another study, Rosero-Bixby and Dow (48) observed, in elders, that various biomarkers, such as blood pressure, creatinine clearance, epinephrine, triglycerides and DHEA-S levels were in healthier levels in low-SES individuals compared to high-SES individuals. It is important to consider that in this study, high-SES individuals are wealthy metro San Jose residents with post primary education; low-SES individuals are poor lowland residents with no education. Goldman et al. analyzed 10 biomarkers in elder population, according to their education level (82). The authors found few differences according to education level, but confirmed higher levels of glucose and triglycerides in high-educated men compared to low-educated men, and highest level of systolic pressure and HbA1c in low-educated women compared to high-educated women.

#### 3.4.5 Obesity

According to the Latin American Study of Nutrition and Health, the prevalence of obesity was 36% among women aged 15 years and older, and 24% among men aged 15 years and older in the period 2014–2015 (83). Herrera-Cuenca’s investigation did not reveal a distinct social pattern for anthropometric measures, such as waist-hip ratio, neck circumference, and waist circumference (83). The authors reported an increase in body mass index (BMI) with higher socioeconomic status (SES), although these findings are inconsistent across gender and age groups, and do not align with the estimated average, suggesting a potential calculation error. Gómez showed that nutritional diversity scores were found to be significantly lower in the low SES (84). Using data from 2005, Mazariegos et al. (85) did not observe a clear social gradient in obesity prevalence, neither in men nor in women. Additionally, using data from the Costa Rican Longevity and Healthy Aging Study (CRELES), Rosero-Bixby and Dow documented a higher prevalence of obesity, high-calorie diet, and abdominal girth among urban populations with high SES compared to non-urban populations with low SES (48). Similar results were found in children: Gamboa-Gamboa et al. documented using the 2016 National School Weight/Height Census that children overweight and obesity prevalence was lower in the poorest districts compared to the wealthiest districts (30.8% versus 37.5%), and in rural districts compared to urban districts (30.8% versus 35.8%) (33).

#### 3.4.6 Diet

Regarding diet, Santamaría-Ulloa et al. (86) showed in urban women (between 2014–2015) that total protein consumption increases with SES, the prevalence of inadequate protein intake was lower in the higher SES group, and that processed meats are consumed mainly by women in medium, followed by low and high SES groups. Monge-Rojas et al. (87) implemented a Traditional Costa Rica Adolescents Diet Score (TCRAD) to assess the main determinants in 2017, positing that traditional diets in Costa Rica are healthier (intake of beans, vegetables, fruits, dairy products). The authors found that adolescents living in rural areas had higher traditional diet scores compared to urban. Nuñez-Rivas et al. (88) assessed diet quality using a scoring system in adolescents. It was found that individuals from high and medium SES groups backgrounds, as well as those with lower levels of education, tended to have better diets compared to those from low SES groups and with higher levels of education. However, these results should be interpreted with caution due to the authors’ reliance on only bivariate statistics.

Herrera-Cuenca et al. observed that calcium intake was significantly higher in high-SES individuals compared to low-SES individuals (83). Iron intake has been found to be similar according to social class in Herrera-Cuenca et al. (83). Lozoff et al. found in children living in an urban community and followed between 1981–1984 and 2000–2002, that infants in the lower SES level (measured by Hollingshead score) were more likely to be in the chronic-iron-deficiency group than in the non-chronic-iron-deficiency group (89).

#### 3.4.7 Cognitive health in seniors

Finally, when it comes to cognitive health and healthy aging, a study reveal that having more years of education was associated with higher cognitive abilities, with a larger effect in rural group (90). Santamaría-García found that the most relevant risk factors negatively affecting cognition were low education, male sex, having mental health symptoms and older age. Education, sex and age were found associated with functional ability (91).

#### 3.4.8 Severe tooth loss

Tooth loss is associated with poor Self-Rated Health (SRH) (92). Using CRELES data, Barboza-Solís et al. showed that adult tooth loss was strongly associated with SES measures, mainly education level, occupational status and subjective economic situation (93). This association was independent of health behaviors, including alcohol, consumption, smoking status, cariogenic diet and psychosocial factors. Fantin et al. also observed, using CRELES data, that adult tooth loss was strongly determined by early SES (94): 72.4% of the people who experienced the most deprived SES during childhood had severe tooth loss at the time of the interview; vs. 43.3% in the most advantaged (94).

## 4 Discussion

Studies at both the individual and district levels in Costa Rica have shown a clear, stepwise association between socioeconomic status (SES) and various health outcomes: as SES decreases, health indicators deteriorate correspondingly.

Mortality is often used to observe health inequalities, as it represents the ultimate health outcome, embodying all economic, social, and biological adversities throughout life (95). While some studies have suggested an absence of health inequalities in Costa Rica (48, 49, 96) overall mortality remains higher among individuals in the least socioeconomically advantaged group compared to the most advantaged (41). Specifically, mortality from respiratory diseases, external causes (such as homicides and car accidents), infectious diseases, endocrine, nutritional, and metabolic diseases, and diseases of the genitourinary system is significantly higher in the poorest districts compared to the wealthiest ones (54). The social gradient in mortality is particularly visible in middle-aged adults (41). In older populations, the gradient is less pronounced, especially among men, where some studies have even observed a reverse social gradient (49).

The observed social inequalities can result from a wide range of factors: unhealthy behaviors (e.g., alcohol consumption, smoking), lack of access to good nutrition and health education, obesity, exposure to occupational and environmental hazards, and limited access to an effective healthcare system (including prevention, timely detection and diagnosis, and high-quality treatments). All these factors are related to an individual’s socioeconomic status, income, wealth, and education level.

Analyzing the social determinants of health allows a better understanding of the reasons why, despite being one of the most inequitable countries in the OECD, health inequalities in mortality are less significant in Costa Rica compared to the US and Europe. For instance, the two main causes of death—diseases of the circulatory system (39–41) and neoplasms (39, 54)—are weakly or not at all associated with SES. Furthermore, while our findings indicate the presence of socioeconomic disparities, they also suggest that health inequalities can be partially mitigated by factors such as the concentration of wealth and the prevalence of risky behaviors, which vary between urban and rural areas. Despite prevalent poverty in both urban and rural areas, individuals in rural areas often exhibit better health outcomes, which may be attributed to their adherence to more traditional, less westernized lifestyles (56). Smoking prevalence is lower among individuals residing in rural areas compared to those in urban areas (80). Some studies also suggest that people living in rural areas have healthier and more traditional diets compared to those in urban areas (87). Alcohol consumption is strongly associated with higher SES (79). This phenomenon could highlight a balancing effect where increased access to resources in urban areas is counteracted by higher engagement in risky behaviors, ultimately resulting in relatively small socioeconomic gradients in health outcomes.

Among women, both smoking and alcohol consumption prevalences are low, regardless of whether they reside in urban or rural settings (79, 80). Consequently, these behavioral factors likely exert small impact on mortality rates. Thus, the observed disparities in women between rural and urban areas are more likely attributable to differences in SES, including wealth (41), income, and education levels.

In men, smoking and alcohol consumption prevalences are lower compared to European countries (28); however, they are still significant enough to yield observable consequences at the geographical level. Notably, in terms of cancer mortality, rural areas exhibit lower mortality rates compared to urban areas for most cancer sites associated with smoking (56).

Regarding access to effective healthcare, the public health system based on EBAIS has demonstrated relatively good results in terms of narrowing inequalities (25). It has been suggested that advancements in access to primary healthcare programs in the 1970s significantly contributed to the decrease in infant mortality, particularly among low SES populations (25). However, disparities persist in accessing diagnosis and/or treatment, as evidenced by social inequalities in survival rates after cancer diagnosis (72). Access to other healthcare services that are inadequately provided by the public health system, such as ophthalmology and dental care, remains highly inequitable (77, 78).

In this review, we included all studies focusing on social inequalities in health in Costa Rica. However, the analysis is limited by the scarcity of available literature. Following the conclusion of the CRELES study, which focused on older adults and completed its last wave in 2012–2013, no subsequent cohort study has aimed to longitudinally monitor the general health of a representative sample of the population. Consequently, the most comprehensive individual-level studies on social inequalities predominantly focus on older population. Furthermore, the absence of a new cohort study hinders the analysis of health outcomes from a life course perspective. To address the scarcity of individual-level data, several studies resort to ecological studies utilizing registries, particularly for assessing mortality, cause-specific mortality, life expectancy and cancer incidence. A notable limitation remains in understanding the association between urbanicity, wealth, and health due to the dearth of studies examining primary social determinants of health beyond smoking, alcohol consumption, obesity, physical activity and dietary habits. Moreover, a significant gap exists as the majority of studies reviewed herein do not incorporate other individual-level variables, such as ethnicity or migration status. Only one study intended to examine health disparities among indigenous populations and did not identify additional mechanisms beyond those previously described in the rural and impoverished territories. However, ethnicity was not measured in this study at an individual level and used indigenous territories as a proxy of ethnicity (96). We did not perform a formal quality assessment, including reporting of results or evaluating the validity and reliability of questionnaires. This decision may be considered a limitation in terms of comparing studies. However, our primary objective was to include all available data and interpret general findings, rather than excluding articles based on quality criteria. Furthermore, we encountered challenges when attempting to adapt existing quality assessment tools to the objectives of this comprehensive literature review, including issues with applicability of certain criteria and difficulties in analyzing ecological studies that were combined with other study designs.

This review highlights social inequalities in health within Costa Rica. These disparities are comparatively less pronounced than those observed in the US or Europe (39, 42). In other middle-income Latin American countries, such as Brazil, Chile, and Mexico, health inequalities are more pronounced than those observed in the Costa Rican context. The extent of these disparities can be explained by structural inequalities shaped by each country’s unique historical, social, economic, political, and healthcare specificities (97–99). The public healthcare system likely contributes partially to the reduced gap between high-SES and low-SES individuals in Costa Rica compared to the US and other Latin American countries. Nonetheless, a significant factor that could explain the inequalities mitigation, is the absence or even reverse gradients in risky health behaviors, such as smoking and alcohol consumption, particularly in rural areas (79, 80). Overall, consumption of alcohol and tobacco is relatively low compared to other OECD countries (28). Consequently, while there exists a relatively modest social gradient in health presently, there is a potential for its amplification with the adoption of more westernized behaviors. Previous evidence has suggested that disadvantaged populations tend to systematically bear the brunt of social, economic, political, and health burdens. However, our findings indicate that Costa Rica exhibits a more nuanced pattern, where individuals from the most disadvantaged sectors do not necessarily experience the cumulative burden seen in other contexts. The Costa Rican example demonstrates that when the prevalence of key risky behaviors is low, health inequalities tend to be reduced. Our study also highlights that in LMICs, socioeconomic status is not the sole factor influencing health inequality. Among disadvantaged populations, we observed significant differences between rural and urban residents. These differences, likely due to less westernized behaviors in rural areas, need to be better studied. More importantly, they necessitate the adaptation of public policies to address the specific needs of diverse populations.

In general, effective strategies to reduce health inequalities may include controlling risky behaviors in the general population and progressing towards universal health coverage with good access to primary healthcare facilities. Prevention policies should combine a population-wide strategy with a vulnerable-group approach to enable proportionate universalism. This approach ensures that interventions are tailored to the level of disadvantage, promoting equity in health outcomes. Future research efforts should prioritize monitoring social inequalities in health, particularly as the population ages. Given Costa Rica’s robust registries, there is an opportunity to delve into the study of rare diseases. Establishing new cohort studies and reinvigorating cross-sectional research initiatives are essential for comprehensively examining mortality trends and prevalent diseases. Individual-level data on health determinants including diet, physical activity, alcohol consumption, and stress exposures, stratified by various socioeconomic measures, are crucial for informing targeted interventions. Incorporating a life course approach within cohort studies can enhance our understanding of how early life events influence health outcomes through various pathways. This approach involves integrating data on SES, environmental exposures, risk factors, and biological measures to uncover associations and inform public health strategies effectively (100).

Costa Rican’s health outcomes are also influenced by the country’s history, political stability, its long-standing democracy, and strong institutional autonomy. The Ministry of Health and the Costa Rican Social Security Fund (CCSS) collaborate with various entities, including the public water company, the national insurance institute, universities, private healthcare services, and municipal and community initiatives. This collaborative network highlights the collective mission of promoting the health and well-being of Costa Ricans.

## 5 Conclusion

The findings highlight social inequalities in health within Costa Rica. Despite being considered one of the most inequitable countries in the OECD, it demonstrates relatively modest social gradients in health compared to other middle and high-income nations. Mortality disparities are shown among middle-aged adults but diminish in the older population. Social patterns in health behaviors can explain the mitigated inequality between rural and urban areas, where rural individuals exhibit lower rates of smoking and alcohol consumption. Additionally, there is an equalizing effect of the Costa Rican universal healthcare system. Disparities persist in accessing diagnosis and treatment, with evidence of social inequalities in cancer survival rates and unequal access to certain healthcare services such as ophthalmology and dental care.

## Data availability statement

The original contributions presented in the study are included in the article, further inquiries can be directed to the corresponding authors.

## Author contributions

CB-S: Conceptualization, Data curation, Formal analysis, Funding acquisition, Investigation, Methodology, Project administration, Resources, Software, Supervision, Validation, Visualization, Writing – original draft, Writing – review & editing. RH: Conceptualization, Funding acquisition, Methodology, Project administration, Resources, Supervision, Validation, Visualization, Writing – original draft, Writing – review & editing. RF: Conceptualization, Data curation, Formal analysis, Investigation, Methodology, Software, Supervision, Validation, Writing – original draft, Writing – review & editing.
